# An Antimethanogenic Nutritional Intervention in Early Life of Ruminants Modifies Ruminal Colonization by Archaea

**DOI:** 10.1155/2014/841463

**Published:** 2014-04-06

**Authors:** Leticia Abecia, Kate E. Waddams, Gonzalo Martínez-Fernandez, A. Ignacio Martín-García, Eva Ramos-Morales, C. Jamie Newbold, David R. Yáñez-Ruiz

**Affiliations:** ^1^INAN, Estación Experimental del Zaidín (CSIC), Profesor Albareda 1, 18008 Granada, Spain; ^2^IBERS, Aberystwyth University, Aberystwyth SY23 3DA, UK

## Abstract

The aim of this work was to study whether feeding a methanogen inhibitor from birth of goat kids and their does has an impact on the archaeal population colonizing the rumen and to what extent the impact persists later in life. Sixteen goats giving birth to two kids were used. Eight does were treated (D+) with bromochloromethane after giving birth and over 2 months. The other 8 goats were not treated (D−). One kid per doe in both groups was treated with bromochloromethane (k+) for 3 months while the other was untreated (k−), resulting in four experimental groups: D+/k+, D+/k−, D−/k+, and D−/k−. Rumen samples were collected from kids at weaning and 1 and 4 months after (3 and 6 months after birth) and from does at the end of the treating period (2 months). Pyrosequencing analyses showed a modified archaeal community composition colonizing the rumen of kids, although such effect did not persist entirely 4 months after; however, some less abundant groups remained different in treated and control animals. The different response on the archaeal community composition observed between offspring and adult goats suggests that the competition occurring in the developing rumen to occupy different niches offer potential for intervention.

## 1. Introduction

Among the ruminal microbiota, members of the domain Archaea, particularly methanogens, are estimated to comprise approximately 0.3–3% of the biomass [1] and play a vital role in microbial fermentation. The majority of the Archaea in the rumen are methanogens, which utilize H_2_ as the energy source to reduce CO_2_ to CH_4_ and provide oxidized reducing factors (e.g., NAD+) to other microbial metabolic pathways [2]. However, the released CH_4_ results in a loss of dietary energy [3] and once released into the environment, methane acts as a potent greenhouse gas, with a much greater effect on climate change than that of carbon dioxide. Consequently, a better understanding of the methanogenic community within the rumen may facilitate the development of strategies to decrease the production of enteric CH_4_.

The literature suggests that, rather than the numbers, it is the structure of methanogen community that drives methanogenesis in the rumen [4]. The diversity of methanogens in the rumen has been shown to depend on environmental factors (i.e., geographical location; [5]) and has strong host specificity [6], which makes it difficult to achieve significant modulation in the adult animal once the rumen is fully developed and the microbial ecosystem established. The developing rumen provides an opportunity to explore means of microbial manipulation. Methanogenic archaea have been found in the undeveloped rumen of lambs well before the ingestion of solid feed begins (2–4 days) and reach levels similar to those in adult animals around 10 days after birth [7, 8]. Skillman et al. [9] suggested that the ewe might be the most important source of microbial inoculation in the young animal. Recently, Gagen et al. [10] reported that the diversity of* mcrA* sequences in the rumen of lambs 17 h after birth was not significantly dissimilar to that found in the mature rumen of conventional 2-year-old sheep. This opens the possibility that methanogens are acquired by ruminants from a very young age and maintained throughout rumen development and life. We have preliminary evidence obtained by DGGE of alteration of the methanogen community that colonizes the rumen in kids treated with an antimethanogen compound (bromochloromethane, BCM) [11]. However, the changes induced in the main archaeal groups and how key species responded remains unknown.

The aim of this work, therefore, was to study whether feeding a methanogenic inhibitor (BCM) during the early life of kids has an impact on the archaeal community that colonizes the rumen and to what extent the impact persists later in life. The effects of adding this compound on CH_4_ production by pure cultures of seven key ruminal methanogens were also investigated. The results from this trial on rumen fermentation and methane production are published in Abecia et al. (2013) [11].

## 2. Materials and Methods

All management and experimental procedures involving animals were carried out by trained personnel in strict accordance with the Spanish guidelines (RD 1201/2005 of 10th October 2005) for experimental animal protection at the Estación Experimental del Zaidín. Experimental protocols were approved (1 October 2010) by the Ethics Committee for Animal Research at the Animal Nutrition Unit.

### 2.1. Animals, Diets, and Experimental Design

Sixteen Murciano-Granadina lactating goats (43 ± 1.7 kg BW) pregnant with two fetuses were acquired at 3 months of pregnancy, kept in individual pens (1.7 m × 1.2 m) with free access to water, and fed alfalfa hay* ad libitum* once a day (in g·kg^−1^ of DM: OM, 880; CP, 214; ether extract, 13.6; NDF, 419: ADF, 244; and ADL, 61) and a supplement 600 g d^−1^ fed twice a day (0900 h and 1500 h) based on (g·kg^−1^): wheat shorts (350), corn shorts (100), corn grain (50), barley grain (160), soybean hulls (90), soybean meal (90), sunflower meal (120), CaO (22), NaCl (3.5), calcium salts (4.5), and tracer minerals and vitamins supplement (10) (in g·kg^−1^ of DM: OM, 893; CP, 170; ether extract, 33.9; NDF, 342; ADF, 142; and ADL, 34.3).

The experimental period commenced when does (D) gave birth, which happened within a period of two weeks. After giving birth, each doe was randomly allocated to 1 of the 2 experimental groups: D+, treated daily with 3 mg of BCM/kg BW divided in two equal doses, and D−, as nontreated control-group but receiving a placebo (10 g of ground oats in cellulose paper and sealed with molasses). Bromochloromethane (99.5%; Aldrich 13,526-7) is a halogenated aliphatic hydrocarbon entrapped in an alpha-cyclodextrin matrix (Alfa Aesar GmbH & Co, A18092) [12]. The BCM formulation was prepared as a dry white powder in 1 to 2 kg batches and contained 10–12% (wt/wt) BCM. The BCM complex was then wrapped in cellulose paper, mixed with 10 g of ground oats, and sealed with molasses. The BCM treatment was given orally twice a day at feeding times (0900 h and 1500 h) to does.

**Figure 1:**
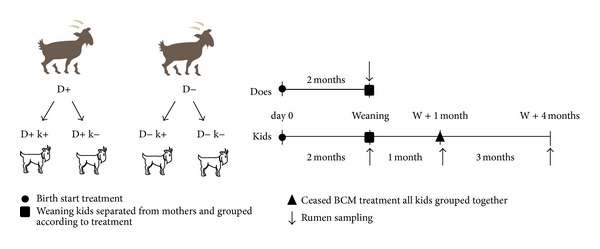
Experimental design and sampling schedule. W = weaning; D+ k+ = treated kids from treated does; D+ k− = untreated kids from treated does; D− k+ = treated kids from untreated does; D− k− = untreated kids from untreated does.

All does gave birth to two kids, one of which remained nontreated (k−), while the other was treated daily with a dose of 30 g/kg BW of BCM as above (k+), thus resulting overall in four kids' experimental groups D+ k+, D+ k−, D− k+, and D− k− (*n* = 8) as illustrated in Figure 1. During the first 2 weeks of life, the BCM formulation was dissolved in 10 mL of water and syringed directly into the mouth of the treated kids twice a day. After 2 weeks, BCM treatment was given orally twice a day at feeding times (0900 h and 1500 h) to kids as described for does. The kids remained with does for 2 months in the same pen with no physical contact with other animals to avoid touching and licking. Kids' weights were registered weekly.

The treatment of kids (k+) lasted for 3 months: 2 months while they remained with the doe and for 1 month after weaning, during which kids were grouped by treatments (D+ k+, D+ k−, D− k+, and D− k−) in four independent pens separated from each other to avoid physical contact. After weaning kids were offered* ad libitum* alfalfa hay and starter commercial compound (g·kg^−1^): wheat shorts (50), corn shorts (50), corn grain (150), oat grain (260), milk powder (190), soybean meal (172), sunflower meal (120), NaCl (3.5), and calcium salts (4.5) (in g·kg^−1^ of DM: OM, 925; CP, 162; ether extract, 35; NDF, 163 and ADF, 78).

At 3 months, all kids from the 4 experimental groups were grouped together in a single pen and BCM treatment ceased (Figure 1). They remained together for another 3 months until the end of the experimental period.

Ruminal content was collected from does 2 months later (2 mo), coinciding with kids' weaning. Ruminal samples were collected from kids three times: at weaning (W) and 1 (W + 1) and 4 months after (W + 4). Samples were taken before the morning feeding using a stomach tube and aliquots were stored at −80°C for further molecular analyses. The results on rumen fermentation and methane emissions from the adult goats and kids have been published recently [11, 13].

### 2.2. Samples Processing and Nucleic Acid Extraction

Samples of rumen digesta for DNA extraction were freeze-dried and thoroughly mixed by physical disruption using a bead beater (Mini-bead beater 8, BioSpec Products, Bartlesville, USA). The extraction was performed from approximately 50 mg samples using the QIAamp DNA Stool Mini Kit (Qiagen Ltd., West Sussex, UK) following the manufacturer's instructions with a modification: a higher temperature (95°C) was used for lysis incubation.

The yield and purity of the extracted DNA were assessed using NanoDrop ND-1000 Spectrophotometer (NanoDrop Technologies, Wilmington, USA).

### 2.3. Pyrosequencing and Sequence Analysis

The hypervariable V6 region of the 16S rRNA gene was amplified using the primer pair, 958F and 1048arcR-major [14]. Primers incorporated 10nt barcode tags and Roche/454 adaptors allowing samples to be pyrosequenced. PCRs were performed, in triplicate, and contained 10x PCR buffer, 10 mM dNTP mix, 10 pmol/*μ*L of forward and reverse primers, 1U FastStart Polymerase, and 1 *μ*L of DNA template which was then made up to 25 *μ*L with molecular biology grade water. The amplification conditions were an initial denaturation step at 95°C for 2 min; 30 cycles of denaturation at 95°C for 30 s, annealing at 55°C for 30 s, and elongation at 72°C for 45 s; and a final extension step at 72°C for 7 min. The size of the PCR products was then checked on a 1% agarose gel electrophoresis. Following this, triplicates were pooled together and products were then purified using the short fragment removal method described by Roche using their GS FLX Amplicon DNA preparation guide and AMPure beads. DNA quantities were calculated using an Epoch Microplate Spectrophotometer (Biotek); based on these quantities, samples at 50 ng/*μ*L were pooled into libraries. These libraries were purified further using E-Gel SizeSelect Agarose Gels (in accordance with Invitrogen's manual).

To determine whether there were any short fragments still present in the library samples, a PCR test was carried out to a total volume of 25 *μ*L, 19.5 *μ*L molecular water, 2.5 *μ*L Fast Start Reaction buffer (make), 1 *μ*L of 10 mM dNTP mix, 0.5 *μ*L Forward Test Primer (make), 0.5 *μ*L Reverse Test Primer (make), 0.5 *μ*L Fast Start Enzyme Blend, and 0.5 *μ*L DNA template (at 2 × 10^8^ molecules). This was run at initial denaturation 94°C for 1 min; 20 cycles of denaturation at 94°C for 1 min, annealing at 60°C for 1 min, elongation at 72°C for 1 min; and a final extension at 72°C for 10 min.

Following this, the PCR test samples were incubated with 0.5 *μ*L of Exonuclease I at 37°C for 30 min. These test PCR samples, along with the libraries and 10-fold dilutions of these libraries, were run on an Agilent Bioanalyser using a DNA High Sensitivity chip. This gave accurate quantification of each library allowing them to be mixed in equimolar amounts to 10^7^ molecules/*μ*L sample.

Finally, the samples were subjected to emulsion PCR and the medium volume (MV) Lib-L Roche protocol was followed. The pooled libraries were then pyrosequenced on a Roche 454 FLX Titanium.

The flowgram (sff) files were converted to fasta DNA (fna) and quality score (qual) file on the 454 cluster and transferred onto a Linux based workstation running the Quantitative Insights into Microbial Ecology (QIIME version 1.5.0) per scripted modules and workflow scripts [15]. Acacia was used for homopolymer error correction [16]. Sequences were filtered to exclude any mismatches to the primer sequence exceeding 6 homopolymer runs or ambiguities and including a minimum sequence length down to 50nt for amplicons. The libraries were split according to the 10nt barcode incorporated into the forward primer. Libraries sequenced on different plates or different lanes of the 454 were pooled.

OTUs were generated by aligning the reads to the GreenGenes database [17] and clustered at 97% sequence identity using the PyNAST tool [15] and UCLUST algorithm [18], respectively. Taxonomic classification was according to the* Basic Local Alignment Search Tool *(BLAST) classifier and a phylogenetic tree was constructed using FastTree [19]. Alpha diversity (i.e., diversity within a sample) indices were generated with the QIIME pipeline. Beta diversity was used to create principal coordinate analysis (PCoA) plots using unweighted UniFrac distances. The Unifrac phylogenetic method [20], which considers phylogenetic lineages and not just shared OTU, was used for community-level comparisons with the trees constructed during the OTU picking script.

### 2.4. Methanogens Pure Cultures

Pure cultures were acquired from DSMZ—German collection of microorganism and cell culture. The species acquired for study were* Methanobrevibacter ruminantium* (strain: DSM 1093; ATCC 35063; JCM 13430),* Methanobrevibacter smithii* (strain: DSM 861; ATCC 35061),* Methanobrevibacter millerae* (strain: DSM 16643; OCM 820),* Methanosphaera stadtmanae* (strain: DSM 3091; ATCC 43021; JCM 11832),* Methanobacterium bryantii* (strain: DSM 863; ATCC 33272; OCM 110),* Methanosarcina barkeri* (strain: DSM 800; JCM 10043; OCM 38) and* Methanomicrobium mobile* (strain: DSM 1539; ATCC 35094; JCM 10551).

Archaea culture was carried out in Hungate tubes with specific medium and growing conditions as specified by DSMZ for anaerobes. Media preparation (119, 120, 161, and 322) is described in detail on their web site (i.e., http://www.dsmz.de/microorganisms/medium/pdf/DSMZ_Medium119.pdf) and was prepared anaerobically, aseptically, and under a gas atmosphere of 80% H_2_ and 20% CO_2_.

For the inoculation, ampoules received with the pure culture were handled within an anaerobic chamber and under a gas atmosphere of 80% H_2_ and 20% CO_2_ as specified by DSMZ (http://www.dsmz.de).

Pure cultures were inoculated in 4 replicates tubes containing 5 mL of the corresponding specific medium. Pressurization with H_2_/CO_2_ gas in an anaerobic chamber was applied to achieve 10^5^ Pa in headspace. For those tubes treated with BCM, the treatment was applied on day 2 (with the exception of* M. mobile* which had a lower growth rate and therefore the treatment had to be applied on day 6). BCM was added at a concentration of 50 *μ*M based on previous* in vitro* and* in vivo* studies carried out at our laboratory [13]. For incubation, tubes were then horizontally placed in a shaking incubator at 37°C at 120 rev min^−1^ in the dark.

Methanogens' growth was followed as CH_4_ production [21, 22]. At days 2, 4, 6, and 8 of incubation (and up to 12 days for* M. mobile*), tubes were analysed for CH_4_ production using a flame ionization-detection GC [23]. In brief, a subsample of 0.5 mL of gas from headspace in each tube was taken and then injected manually in the GC (HP Hewlett 5890, Packard Series II, Waldbronn, Germany) using a 1 mL SampleLock syringe (Hamilton, Nevada, USA). The concentration of CH_4_ was determined using a standard curve generated by injecting different volumes of 99.9% pure CH_4_ before and after the injection of samples.

### 2.5. Statistical Analyses

Data were analyzed using the SAS PROC MIXED procedure [24]. The statistical model used included the effects of BCM treatment to does, kids, and the doe × kid interaction as fixed effects and animal effect was considered random. When doe × kid interaction was significant (*P* < 0.05), differences between treatment means were evaluated using the “pdiff” option of the “LS means” statement in the MIXED procedure of SAS and declared significant at *P* < 0.05. A tendency was considered when *P* values were <0.1. The significance of the effects on the distribution of the different archaeal groups is included in the text.

## 3. Results

### 3.1. Archaeal Community Structure in the Rumen of Does

**Figure 2:**
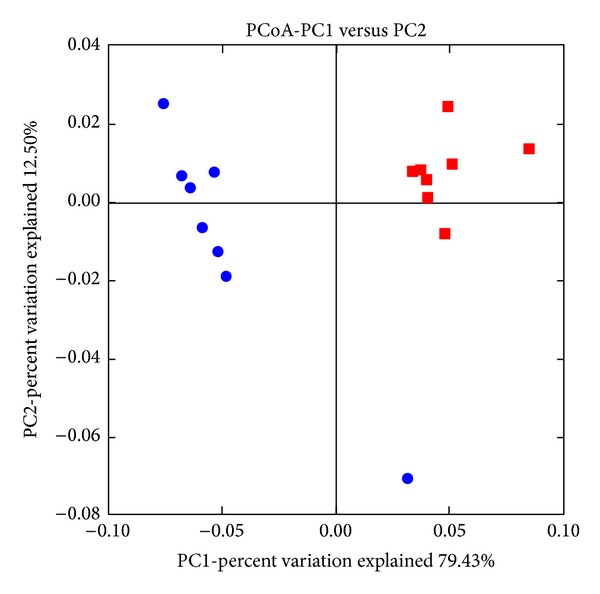
Principal component analysis of rumen methanogenic archaea profiles in does: blue circles = D+ and red square = D−.

An average of 7557 sequences per sample was used for analysis after quality control. Taxonomic analysis of the archaeal community using the BLAST classifier (Table 1) revealed that Methanobacteriales order was the most representative in the rumen of both groups of does (D+ and D−). The treatment did not have any significant effect in the relative abundance of the family Methanobacteriaceae, althougha numerical increase and decrease, respectively, of* Methanobrevibacter *and* Methanosphaera *was observed. Other minor groups (Crenarchaeota, Methanomicrococcus, and Thermoplasmata) were also detected in low proportion (less than 0.3%) in the control group that were not detected in D+ animals. These differences were illustrated in the PCoA (Figure 2) that showed two separate clusters according to does' treatment.

As shown in Table 3, the treatment with BCM to D resulted in higher number of observed species (OS), Chao and Shannon index (H).

### 3.2. Archaeal Community Structure in Kids at Weaning (W), 1 (W + 1), and 4 (W + 4) Months Later

**Figure 3:**
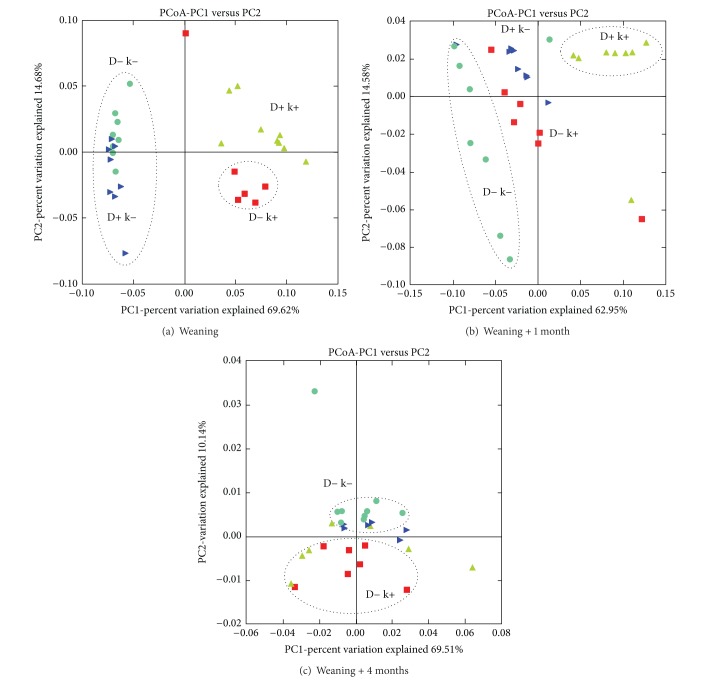
Principal component analysis of rumen methanogenic archaea profiles at (a) weaning, (b) 1 month after, and (c) 4 months after weaning. Experimental groups: up-pointing pale green triangle = D+ k+, right-pointing blue triangle = D+ k−, green circles = D− k−, and red square = D− k+. D+ k+ = treated kids from treated does; D+ k− = untreated kids from treated does; D− k+ = treated kids from untreated does; D− k− = untreated kids from untreated does.

An average of 4414, 4922, and 4713 sequences per sample was used at W, W + 1, and W + 4, respectively, for analysis after quality control.  Table 2 shows the taxonomic analysis of the archaeal community using the BLAST classifier. At W a significant (*P* ≤ 0.009) effect of treatment applied to the kids was observed on the abundance of* Methanobacterium*,* Methanobrevibacter*, and* Methanosphaera. *The k+ kids presented greater and lower abundance, respectively, of* Methanobrevibacter *and* Methanosphaera*. On the other hand,* Methanobacterium* was significantly greater in D− k− kids compared to the other three groups in which either the doe or kid was treated. Other minor groups were detected only in treated kids (*Nitrososphaera* in D+ k+ and* Halobacterium* in D− k+). The PCoA plot of the archaeal community structure (Figure 3(a)) segregated the samples according to the treatment received by kids (D+ k+ and D− k+ were clearly separated from D+ k− and D− k−) by the vertical axis, which explained 69.6% of the variability.

At W + 1, the BCM treatment applied to kids significantly affected the abundance of* Methanobrevibacter, Methanosphaera, Methanoplanus*, and* Thermoplasmata, *although the effect was not always in accordance with what observed at W. Also, there was a significant interaction with the treatment received by does on the abundance of* Methanobrevibacter* and* Methanosphaera* as only k+ kids raised by D− does showed differences as compared to k− kids. The PCoA plot of samples collected at W + 1 (Figure 3(b)) differentiated D− k− and D+ k+ groups, while D− k+ and D+ k− animals were spread in between D− k− and D+ k+.

At W + 4, when all kids were grouped together and BCM treatment had ceased 3 months earlier, the taxonomic analysis of the archaeal community revealed no significant effect of BCM treatment applied to kids. However, the abundance of* Methanobrevibacter *and* Methanosphaera* remained, respectively, numerically lower and higher in D− k+ animals compared to the other three groups. Interestingly, there was a significant interaction (*P* < 0.001) between treatment applied to kids and does on the abundance of* Thermoplasmata* group, which resulted in lower values in D− k+ kids. Also, sequences belonging to phylum Crenarchaeota were only detected in kids that had not been treated in early life (D− k− and D+ k−). The PCoA plot (Figure 3(c)) showed a less evident segregation of experimental groups according to the community structure, although D+ k+ and D− k+ samples were separated by a component that explained 12%.

In terms of diversity, Chao (species richness), observed species (unique OTUs), and Shannon index in the rumen contents of the four experimental groups of kids at weaning, 1 month, and 4 months after weaning are presented in Table 3. Overall, the treatment applied to k and D resulted in significant alteration of diversity indices, including k × D interaction. However, we could not evidence a clear pattern of the effects when the four treatments were compared and the sequence of the three collection times analyzed.

### 3.3. Methanogens Pure Cultures

**Figure 4:**
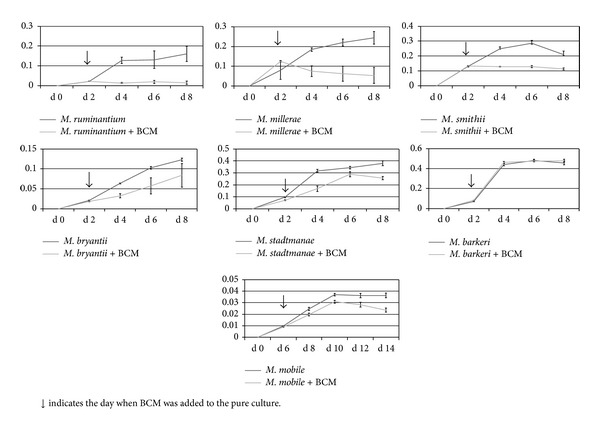
Assessment of effect of adding BCM on the growth (mL CH_4_/mL gas) of seven Archaea strains (*Methanobrevibacter millerae, Methanobacterium smithii, Methanobacterium bryantii, Methanobrevibacter ruminantium, Methanosarcina barkeri, Methanosphaera stadtmanae*, and* Methanomicrobium mobile*) studied in this experiment.

The growth of the seven species of Archaea* in vitro* was monitored over 8 days (12 days for* M. mobile*) and the pattern obtained from all strains showed important differences (Figure 4). As a common feature, and with the exception of* M. mobile*, a steady state level was achieved around 8 days postinoculation of cultures, probably due to the limitation of some nutrients or saturation with metabolic end-products.

The monitoring of culture growth showed that BCM had a potent inhibition effect on* M. ruminantium, M. millerae, M. smithii, *and* M. bryantii*, with* M. ruminantium* being the most sensitive strain to the treatment. BCM slightly inhibited the growth of* M. stadtmanae* and* M. mobile* and did not affect that of* M. barkeri*.

## 4. Discussion

The hypothesis to test in this work is to what extent the microbial population that first establishes in the rumen would have an impact on the microbial ecosystem later in life. We have observed that a simple feeding regime (forage versus concentrate) applied during the early life of lambs modified the bacterial population colonizing the rumen and this effect persisted over 4 months [25]. We have recently reported that feeding BCM to kids and does altered the rumen fermentation pattern and reduced methane emissions [11, 13]. This is reflected in more propionic type of fermentation which results in the persistency of lowered methane emissions in the offspring treated in early life and raised by treated does (D+ k+). The present work aims to provide more insight into the changes occurring in the archaeal community during colonization of the rumen and the persistency of the effects in later life of the animal.

BCM is one of the most effective inhibitor that reduces methane production by interfering with the cobamide-dependent methyl transferase step of methanogenesis [26, 27]. When BCM is entrapped in cyclodextrin and fed to ruminants, it causes a sustained inhibition of methane production [12, 28, 29]. Moreover,* in vitro* incubations in continuous culture system demonstrated that BCM significantly reduced methane production (85–90%), whereas there was no effect on total volatile fatty acids production, true degradability of feed, and the efficiency of microbial protein synthesis [30]. It is predicted that H_2_ gas would accumulate in the rumen when methanogenesis is strongly inhibited by suppression of the growth of ruminal methanogens [31]. Mitsumori et al. [32] concluded that in goats the inhibition of methanogenesis by >80% substantially increased ruminal H_2_ concentration without affecting dry matter intake and feed digestibility. To what extent this excess of hydrogen drives the shift in methanogens' diversity when the rumen is developing is discussed below. Recently, the treatment with BCM has been associated also with a change in the structure of the bacterial community [32]. The increase in the relative abundance of* Prevotella* could be related to H_2_ accumulation due to decreased methane production [30]. Likewise, Mitsumori et al. [32] reported the negative effect on* R. albus *as a result of treating goats with BCM due to the high sensitivity to high partial pressure of H_2_. This decrease might be compensated by greater abundances of other fibrinolytic bacteria such as* F. succinogenes *that does not produce H_2_ and is not susceptible to H_2_ accumulation.

### 4.1. Methanogenic Archaeal Diversity

Methanogenic archaea comprises a diverse and complex group that plays an essential role in the rumen; however, its biodiversity still remains largely unknown as in other microbial ecosystems [33]. Recent developments in culture independent molecular techniques have led to identifying a wider range of species including those from the methanogenic community that are yet uncultivable using traditional techniques. However, results differ across different studies.

Based on our previous work [11], DGGE banding profile of the same samples as used here showed that BCM treatment induced differences in archaeal community structure in the developing rumen of kids at W and W + 1. However, at W + 4 the treatment applied to kids only promoted different archaeal community structure in kids raised by D+ does, suggesting that the treatment received by the does was the most influential factor on the archaeal community structure. On the other hand, although biomass of methanogens in the rumen of kids at W was equivalent to that in adult goats, the impact of BCM treatment on biomass was variable at W, W + 1, and W + 4, and not correlated with the patterns observed for CH_4_ production. Therefore, it appears necessary to identify what archaeal groups vary among treatment in order to hypothesize which ones would be beneficial to promote in the developing rumen.

In the present work,* Methanobrevibacter* accounted for 87% to 92% of total archaeal sequences in the rumen of does, while* Methanosphaera *represented between 7% and 11%. In a recent study, Kim et al. [33] reported that* Methanobrevibacter, Methanomicrobium, *and* Methanosphaera* accounted, respectively, for 50%, 15%, and 13% of all archaeal analysed sequences in the rumen. The overall predominance of* Methanobrevibacter* spp. is in agreement with the abundance reported in different studies [1].

The response by different archaeal species to BCM may be associated with the H_2_ partial pressure in the environment as a result of inhibition of methanogenesis [34]. In this study, the proportion of* Methanobrevibacter *group, which has been shown to be highly sensitive to the excess of H_2_, did not change in does. However, in k+ kids there was a significant increase at W and W + 1 month and a numerical decrease in D− k+ kids at W + 4. A similar comparison can be made for the* Methanosphaera* group. This suggests that the scenario observed in adult animals might not apply in the developing rumen. Rumen development is an important factor determining early solid feed intake and performance in ruminants [35]. The established rumen microbiome in the young animal lays a solid foundation for the transition from the pre- to the ruminant state, a critical stage for later life. Many major functional groups of ruminal microorganisms—including cellulolytic and sulfate-reducing bacteria, as well as other hydrogen-utilizing species, such as acetogenic bacteria—become established in the rumen within the first week of life [8, 36]. The role played in this experiment by the acetogenic bacteria might partly explain the different response observed in does and kids treated with BCM. The early establishment of acetogenic bacteria underlines the competition that exists between different H_2_ utilizing species within the rumen. Morvan et al. [8] observed that acetogens did colonize the rumen of lambs before methanogens did. Also Faichney et al. [37] reported that lambs reared in isolation from their dams produced 30–40% less methane than conventional animals and H_2_ appeared to be channeled via reductive acetogenesis. The combination of acetogenic bacteria being the dominant hydrogenotrophs in the developing rumen and the treatment with BCM applied in early life may have resulted in a peculiar methanogenic community that otherwise would not have been developed if the treatment was applied in the adult animal when acetogens are less likely to occupy the niche [38]. This hypothesis would need to be confirmed by monitoring the establishment and response of the acetogenic community in future studies.

Regardless of the different response in does and kids at weaning, a significant interaction between the treatments applied to the kid depending on whether the doe was treated or not suggests that the dam (or any other adult animal within the flock in contact with the offspring from birth) plays a key role in the microbial colonization of the developing rumen. This opens the possibility of intervention through treating mothers and potentially might have implications in practical farming management and feeding, especially in intensive systems where the offspring is taken away from the dam straight after birth.

Furthermore, as described in previous work, D+ k+ animals from this experiment remained lower methane emitters four months after weaning [11]. Although the main effects of applying BCM on the archaeal community structure on the major groups detected in this study (*Methanobrevibacter* and* Methanosphaera*) did not persist in kids at W + 4, future studies should use more sensitive techniques to detect other known less abundant but yet important groups such as* Methanosarcina*. In this line, the* Thermoplasmata* group (accounting for a low proportion of the total community) remained different at W + 4. It cannot be ruled out the possibility of some species to make a greater contribution to methane emissions than their abundances suggest by having greater transcript* mcrA* gene [39]. A recent work, using DNA and RNA derived* 16S* rRNA analysis [40], has shown that* Methanobrevibacter* species despite being numerically predominant only contributed to a third of the RNA-derived* mcrA* sequences, while a less abundant species (*M. luminensis*) represented the majority and may contribute highly to methane formation. Indeed, Poulsen et al. [41] reported in a metatranscriptomic survey that* Thermoplasmata 16S* rRNA and methylcoenzyme M reductase (*mcr*) transcripts decreased concomitantly with mRNAs of enzymes involved in methanogenesis in the rumen of cows treated with rapeseed oil, while the major archaeal groups were not affected.* Thermoplasmata* is a novel group of methylotrophic methanogens in the bovine rumen that use methylamines as their major energy and carbon sources. They have been recently associated with reduced methane emissions in cattle treated with rapeseed oil. It has also been suggested that their contribution to methane formation might be underestimated by the numerical abundance [42] and it appears that they are the dominant methanogen population in the rumen of Qinghai-Tibetan sheep. In the same line, the phylum Crenarchaeota, another unusual minor archaeal group, was detected only in nontreated kids at W + 4. Sequences of rDNA from non-Thermophilic-Crenarchaeota and Thermophilic-Crenarchaeota in the rumen were first described by Madigan and Martinko [43], although no known role has been described in this environment. Initially, the Crenarchaeota were thought to be sulfur-dependent extremophiles but recent studies have identified characteristic Crenarchaeota environmental rRNA indicating that this group may be the most abundant archaea in the marine environment [44]. Thus, it seems necessary to complement the structural analysis of the ecosystem with functional assays (transcriptomic analysis and/or pure cultures) to fully understand the importance of some theoretically minor species with regard to methane production.

Zhou et al. [4] indicated that the methanogenic communities in animals with low feed efficiencies were more diverse than those in efficient ones and the prevalence of* M. stadtmanae* and* Methanobrevibacter* sp. were around 2 times higher in inefficient animals. This agrees with our results showing that some species from another group such as Methanomicrobiales might take over when methane emissions are substantially dropped.

### 4.2. Methanogens Pure Cultures

As discussed above, the lack of direct relation between methanogen numbers and methane production has been ascribed to differences in the composition in archaeal species present in the rumen with potentially different methane production rates [4]. Mitsumori et al. [32] reported a half-log reduction in the normal methanogen population correlated with >50% reduction in methane, indicating that the relative methanogenic activity of different archaeal species in the rumen plays a greater role in determining methane output than absolute number of methanogens. Therefore, any study on methanogenesis inhibition should account for the effect on a representation of methanogenic archaea species within the rumen and not only on those that are believed to be numerically dominant.

The first five steps of the hydrogenotrophic pathway result in the sequential reduction of CO_2_ by electrons sourced from H_2_ to form N_5_-methyl-tetrahydromethanopterin methyl transferase [44]. The methyl group is then transferred to coenzyme M via the action of methyl-H_4_MPT:CoM-methyltransferase which is encoded by the* mtr *gene cluster [45]. BCM interferes with the cobamide-dependent methyl transferase step. The* mcr-A* form is thought to be present in all methanogens, whilst the presence of* mcr *II form has only been demonstrated in members of the orders Methanobacteriales and Methanococcales [46]. Kong et al., [47], suggested that* M. ruminantium*-related methanogens play an important role in ruminal methanogenesis as they comprised about half of the total methanogen cells in the rumen. However, this is in contrast with recent observations as discussed above [40]. In our pure culture experiment,* M. ruminantium* was highly sensitive to BCM probably because its genome does not encode a methyl coenzyme reductase II (*mcr* II or* mtr*) system. This cluster of genes is found in some methanogens, and it encodes an iso-enzyme of the methyl CoM reductase I enzyme and is differentially regulated during growth [36] to mediate methane formation at high partial pressures of H_2_. However, H_2_ does not accumulate to high levels in the rumen, so it appears that* M. ruminantium* has adapted its lifestyle for growth under low levels of H_2_ using the* mcr* I system only [45]. We hypothesize that the same occur with* M. millerae *and* smithii. *However, the* in vivo* results do not agree with greater sensitivity of* Methanobrevibacter in vitro*, which suggests that species, other than the 3 cultivated in this group, might be highly involved.

Comparison of methyl coenzyme reductase II (*mcr* II or* mtr*) genes from species within the order Methanobacteriales shows that* M. smithii* and* M. stadtmanae* share* mtr* genes with* M. ruminantium*. However, many of the differences in gene sequences from* M. stadtmanae* and* M. smithii* encode for surface proteins that are likely to mediate interactions within its environment and possibly with other rumen microorganisms [45]. This might explain a less acute inhibition of growth by these two species as it occurred for* M. ruminantium*.

The resistance of* M. barkeri* to BCM might be associated with its metabolically versatile lifestyle. More than one methylcobamide:CoM methyltransferase from* M. barkeri* has been characterized [48–50]. Methanosarcina are the only known anaerobic methanogens to produce methane using all three known metabolic pathways for methanogenesis. This microorganism could be one of the nontargeted methanogens that would expand their populations to fill the niche previously occupied by methanogens sensitive to BCM. Unfortunately the set of primers used in this work for pyrosequencing did not allow us to detect enough sequences from the Methanomicrobia to be compared with pure culture growth of* M. barkeri* and* M. mobile*. Based on this, the next step to support our hypothesis would be to study the abundance of* M. barkeri* in the experimental groups and using more powerful sequencing tools.

In conclusion, our results show that nutritional intervention during the early life of ruminants with an antimethanogen compound results in a modified structure of the archaeal community colonizing the rumen. The effects seem to be influenced by the treatment applied to the doe and, in relation to the major archaeal groups detected in this study, it does not persist 3 months after the treatment stops. However, some less abundant unknown archaeal groups could play a key role in the persistency of the lowered methane emissions in treated offspring. The different response of the archaeal community observed between offspring and adult goats suggests that the competition occurring in the developing rumen to occupy different niches offer potential for intervention.

## Figures and Tables

**Table 1:** Effect of bromochloromethane (BCM) treatment of does on relative abundance (% of total sequences) in the rumen contents at weaning (W).

Taxonomy (%)	D−	D+	SED	*P* value
No blast hit		0.3		
p_Crenarchaeota	0.1			
p_Euryarchaeota				
c_Methanobacteria				
o_Methanobacteriales				
f_Methanobacteriaceae; g_*Methanobrevibacter *	87.7	92.1	1.86	0.24
g_*Methanosphaera *	11.6	7.6	1.72	0.24
f_Methanosarcinaceae; g_*Methanimicrococcus *	0.3			
c_Thermoplasmata				
o_E2				
f_WCHD3-02	0.3			

p: phylum, c: class, o: order, f: family, g: genus; SED: standard error of difference.

Does treated (D+) or untreated (D−) with bromochloromethane over lactation period.

**Table 2:** Effect of bromochloromethane (BCM) treatment of does and kids on relative abundance (% of total sequences) at weaning, 1 month, and 4 months later in the rumen of kids.

	BCM treatment				SED	BCM *P* value		
	D− k+	D+ k+	D− k−	D+ k−		kid	Doe	k × D
Weaning								
No blast hit			0.2	0.1				
f_Methanobacteriaceae			0.1	0.1				
g_*Methanobacterium *	0.2	0.1	1.7	0.1	0.221	0.0094	0.001	0.012
g_*Methanobrevibacter *	82.2	80.8	71.8	63.3	3.140	0.0002	0.079	0.24
g_*Methanosphaera *	17	18.2	25.9	36.2	3.110	0.0002	0.052	0.13
f_Methanococcaceae								
g_*Methanococcus *	0.2							
f_Methanosarcinaceae		0.1						
g_*Methanimicrococcus *		0.2	0.2	0.1				
c_Thermoplasmata								
f_WCHD3-02	0.1	0.4	0.2	0.1	0.217	0.28	0.23	0.13
Weaning + 1 month								
No blast hit	0.2	0.1						
f_Methanobacteriaceae	0.1		0.1	0.1				
g_*Methanobrevibacter *	78.0	91.4	94.7	90.4	3.162	0.025	0.44	0.0005
g_*Methanosphaera *	21.4	8.1	5.1	9.5	3.153	0.032	0.48	0.0005
f_Methanomicrobiaceae								
g_*Methanoplanus *	0.1	0.2						
c_Thermoplasmata								
f_WCHD3-02	0.1	0.2	0.1					
Weaning + 4 months								
No blast hit		0.1	0.1	0.1				
p_Crenarchaeota			0.2	0.2				
p_Crenarchaeota; c_MCG			0.3	0.4				
f_Methanobacteriaceae		0.1		0.1				
g_*Methanobacterium *	0.3	0.2	0.1	0.1	0.096	0.12	0.42	0.73
g_*Methanobrevibacter *	80.5	85.5	83.4	83.7	2.322	0.78	0.35	0.57
g_*Methanosphaera *	19	13.3	14.2	14.8	2.160	0.72	0.44	0.22
f_Methanomicrobiaceae								
g_*Methanoplanus *			0.1					
f_Methanosarcinaceae								
g_*Methanimicrococcus *		0.1	1	0.1				
c_Thermoplasmata								
f_WCHD3-02	0.2	0.5	0.6	0.4	0.088	0.32	0.69	0.0006

p: phylum, c: class, o: order, f: family, g: genus; D+ k+: treated kids from treated does; D+ k−: untreated kids from treated does; D− k+: treated kids from untreated does; D− k−: untreated kids from untreated does.

Effect of BCM (bromochloromethane) treatment on kids (k), Does (D), and D × k interaction (*n* = 8).

SED: standard error of difference.

**Table 3:** Effect of bromochloromethane (BCM) treatment of does and kids on diversity (Chao; observed species, OS; and Shannon, H) of the rumen archaeal community in does at weaning and kids at weaning, 1 month, and 4 months later.

Indices		D−	D+		SED	BCM *P *value		
						Doe	kid	D × k
OS		99.7	129.3		12.01	0.027		
Chao		135.5	182.5		18.62	0.024		
H		4.89	5.43		0.141	0.002		

	D− k+	D+ k+	D− k−	D+ k−				

Weaning								
Chao	90.6	127.4	166.6	182.7	7.370	<0.0001	<0.0001	0.050
OS	66.4	104.9	127.1	131.6	6.647	<0.0001	<0.0001	0.001
H	4.61	4.82	5.82	5.85	0.141	0.21	<0.0001	0.34
Weaning + 1 month								
Chao	195.5	172.8	183.8	111.9	19.19	0.001	0.011	0.076
OS	144.0	131.6	133.7	72.0	12.84	0.0003	0.0006	0.010
H	6.10	4.55	5.48	5.18	0.219	<0.0001	0.96	0.0003
Weaning + 4 month								
Chao	87.9	117.7	191.4	101.8	6.175	0.0115	0.0005	<0.0001
OS	72.8	86.8	117.1	85.1	4.531	0.16	0.0025	0.001
H	4.63	4.64	4.66	4.70	0.126	0.0739	0.0521	0.092

D+ k+: treated kids from treated does; D+ k−: untreated kids from treated does; D− k+: treated kids from untreated does; D− k−: untreated kids from untreated does.

Effect of BCM treatment of doe (D), kid (k), and D × k interaction (*n* = 8).

## Abbreviations

BCM: Bromochloromethane
CH4: Methane
k: kids
D: Does
W: Weaning.
